# Biomimetic Materials for Skin Tissue Regeneration and Electronic Skin

**DOI:** 10.3390/biomimetics9050278

**Published:** 2024-05-07

**Authors:** Sol Youn, Mi-Ran Ki, Mohamed A. A. Abdelhamid, Seung-Pil Pack

**Affiliations:** 1 Department of Biotechnology and Bioinformatics, Korea University, Sejong-Ro 2511, Sejong 30019, Republic of Korea; youseul0419@korea.ac.kr (S.Y.); mohamed42@korea.ac.kr (M.A.A.A.); 2 Institute of Industrial Technology, Korea University, Sejong-Ro 2511, Sejong 30019, Republic of Korea; 3 Department of Botany and Microbiology, Faculty of Science, Minia University, Minia 61519, Egypt

**Keywords:** biomimetic, wound healing, wound dressing, E-skin, smart bandage, nature inspired, real-time monitoring, nanostructure, diatom

## Abstract

Biomimetic materials have become a promising alternative in the field of tissue engineering and regenerative medicine to address critical challenges in wound healing and skin regeneration. Skin-mimetic materials have enormous potential to improve wound healing outcomes and enable innovative diagnostic and sensor applications. Human skin, with its complex structure and diverse functions, serves as an excellent model for designing biomaterials. Creating effective wound coverings requires mimicking the unique extracellular matrix composition, mechanical properties, and biochemical cues. Additionally, integrating electronic functionality into these materials presents exciting possibilities for real-time monitoring, diagnostics, and personalized healthcare. This review examines biomimetic skin materials and their role in regenerative wound healing, as well as their integration with electronic skin technologies. It discusses recent advances, challenges, and future directions in this rapidly evolving field.

## 1. Introduction

### 1.1. Physiological Stages of Wound Healing

**Figure 1:**
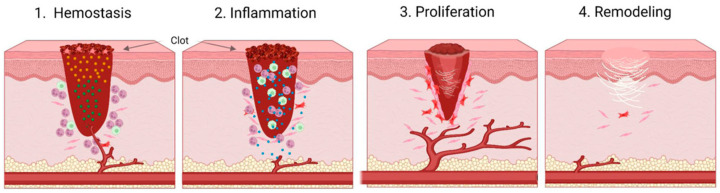
Schematic of the stages of wound healing. It goes through four phases: hemostasis, inflammation, tissue regrowth, and remodeling. The processes that occur at each phase are distinct. Reproduced with permission from [3], Copyright 2023 Frontiers (CC BY 4.0).

A wound is a break in the skin or mucous membrane caused by physical or mechanical damage [1]. When a wound occurs, it undergoes a complex process to repair the damaged tissue. This process is divided into four main phases: hemostasis, inflammation, proliferation, and tissue remodeling (Figure 1) [2]. Each phase involves different responses and occurs over a specific period of time.

Firstly, hemostasis is a coagulation cascade reaction in which blood vessels constrict when a wound is formed and form a blood clot containing platelets and fibrin [4]. The initial step in the process is vasoconstriction, which is triggered by the release of endothelin-1 from smooth muscle, endothelial cells, and platelets. Its purpose is to reduce blood flow and prevent blood loss. Following vasoconstriction, adhesion occurs. This is caused by the adhesion of platelets to the subendothelial layer, which induces aggregation and activation, leading to the formation of a platelet plug. Collagen, laminin, and subendothelial elements exposed to blood due to a vascular injury assist in this process [5]. Platelets adhere to the site of injury and prevent bleeding from the damaged blood vessel by forming a clot, typically within a few hours [6,7].

The subsequent stage of inflammation involves the discharge of proteolytic cytokines into the wound. This process is instigated by invading immune cells and typically lasts between one to three days [8,9]. Macrophages support the healing process by promoting cell proliferation and protein synthesis through the release of growth factors [10]. Additionally, during this period, exudate is produced due to vasodilation. Exudate, which is essentially a fluid that maintains a moist environment, has the potential to promote wound healing. However, in chronic wounds, it can actually cause inflammation or infection [11,12]. With this influence, in chronic wounds, the inflammatory response can persist for an extended period, creating opportunities for bacterial infection [13].

The proliferative phase starts with the proliferation of fibroblasts and myofibroblasts, which can take between 4 and 21 days [14]. At present, fibroblasts create a matrix that facilitates wound contraction to mend the wound edges [15]. Fibroblasts are recruited to the wound by platelet-derived growth factor and transforming growth factor-β. They proliferate and produce matrix proteins, collagen, and proteoglycans. This creates an important interaction between fibroblasts and the extracellular matrix, which is involved in the regulation of further synthesis and remodeling. After the extracellular matrix (ECM) has formed, granulation tissue develops, enabling angiogenesis and the migration of endothelial cells. These cells then multiply and mature [16,17]. Epithelialization leads to the final stage of tissue remodeling, where the cells transform into epidermal cells.

The final stage of the wound healing process lasts between 21 days and a year. During this phase, the granulation tissue is covered by a cellular barrier [18]. The remodeling process can result in scarring as a result of cell death and efforts to repair the structure [3]. The wound matrix’s nature changes depending on the scar. In immature scars, collagen fibers are disorganized, and the matrix grows into thick fibers. The duration of the remodeling process depends on the intensity of the wound disruption, highlighting the importance of rapid wound healing [19].

### 1.2. Advancement in Wound Management Techniques

Wound healing is a complex process that involves multiple reactions. Many factors make complete healing difficult [20]. In addition, incorrect healing can result in chronic wounds [21,22]. Exposure to this can result in a range of health issues, including inflammation, burns, diabetes, infections, and fibrosis [23,24,25,26,27]. Indeed, in the context of diabetic wounds, the process of wound regeneration is impeded by the deleterious effects of bacterial infections on the inflammatory milieu. This, in turn, gives rise to a state of inadequate healing, which can culminate in the necessity of limb amputation [28]. Therefore, there is a growing interest in wound management techniques that promote fast and effective healing [29]. In general, wound care entails applying various types of dressings [30]. Wound dressings serve to shield the wound from external factors, preventing further harm and microbial infection [31]. Wound dressings create a moist environment to accelerate wound healing [32]. Creating a moist environment within 48 h of injury generally achieves a faster phase of tissue remodeling [33]. However, creating a moist environment at the wound site and ensuring adhesion can be challenging when using traditional wound dressings [34]. Choosing the appropriate dressing for wounds can be challenging due to the wide variety of options available [35]. Furthermore, contemporary wound dressings solely offer protection from external factors and do not facilitate skin regeneration or healing processes. This highlights the necessity for more advanced wound care technologies [36,37,38]. There are numerous techniques for effective wound management. The process of wound healing, hemostasis, antimicrobial treatment, adhesion, and the ability to monitor the wound’s condition are all complex [39,40]. These technologies can accelerate wound healing in complex wounds and achieve wound contraction without scarring [41,42,43,44]. In recent years, there has been a growing need for improved wound management techniques due to factors such as an aging population and an increase in patients with diabetes [45,46].

### 1.3. Concept of Biomimetic Materials

Biomimicry refers to the imitation of living organisms or natural processes. It involves biologically inspired design or the derivation of ideas from nature and is commonly known as biomimicry [47]. The term ‘bios’ originates from the Greek word for life, while ‘mimesis’ means imitation or likeness [48]. In 1957, Dr. Otto Schmitt developed a physical device that imitates the electrical activity of nerves [49]. Biomimicry has numerous applications in everyday life, as exemplified by Velcro tape and microfiber swimwear. Swiss engineer Georges de Mestral invented Velcro tape in the late 1940s, inspired by the ability of salamanders to attach to clothing and fur. The surface of a salamander has hundreds of tiny hooks, which he utilized to facilitate attachment and detachment [50]. Tiny bumps on a shark’s skin reduce drag and improve thrust. The creation of a full-body swimsuit with microscopic bumps is one of the best examples of biomimicry [51,52].

Due to these numerous advantages and uses, its use is also gaining attention in the field of wound care technology. Some representative examples include the imitation of the structure of the human body (skin), the imitation of animals and plants, and the imitation of nanostructures. The imitation of the original normal structure is the most basic way to repair skin damage. It can display surfaces, topography, and microstructures like real skin and promotes cell proliferation [53]. Furthermore, mussels are capable of firmly attaching to surfaces due to the presence of adhesive proteins. This adhesion to the wound area has been demonstrated to facilitate rapid wound healing [54].

In this way, biomimicry is utilized in various technologies to enhance their performance, including wound care technologies. The application of biomimicry in the field of life sciences is expected to result in the development of improved wound care technology by combining biotechnology, medicine, and nanotechnology with technologies inspired by design and nature in living organisms [55].

### 1.4. Electronic Skin

Electronic skin (E-skin) is an electrical device or system that mimics the mechanical elasticity, conformability, self-healing, and biocompatibility of skin, as well as its advanced sensory capabilities through mechanoreceptors [56]. Recent advancements in flexible electronics and soft materials science have led to the rapid development of E-skins for wearable health monitoring, medical devices and implants, artificial prosthetic skin, humanoid robotics, and human–machine interface applications [57,58,59,60,61]. This indicates the significant potential of E-skins as multifunctional flexible electrical platforms [62].

Designed to mimic human skin, wearable smart patches non-invasively continuously monitor physiological and biochemical information around a wound [63,64]. By providing real-time data, wound conditions can be detected and assessed early, allowing for timely intervention for treatment [58,65]. Remote wound management via smartphone applications can also facilitate early intervention by regularly scanning wounds at home, allowing for timely adjustments to wound care [66,67]. Furthermore, researchers are developing systems that integrate wound condition monitoring with on-demand drug delivery [68,69]. This will enable targeted treatment by releasing drugs exactly when they are needed.

## 2. Biomimetics Skin for Wound Care Techniques

The bioenvironment employs biomacromolecules that are genetically controlled to shape tissues and regulate biological function and physical performance [70]. Mimicking the biological environment is essential because it controls the functioning of many tissues. Among these, mimicking the human body, including the skin, has been the preferred method in many biomimetic studies because it closely resembles the skin in its normal state. The tissues and organs of the human body, including the skin, are composed of cells, biomolecules, and the extracellular matrix. Therefore, mimicking them or mimicking the structure or mechanism of the skin is crucial [71]. The imitation of skin is achieved using a variety of technologies, including 3D printing and layer-by-layer technology. Electrospinning is a technology that produces nano-sized fibers by applying a strong electric field to a polymer solution or melt [72]. The electrospinning technique can mimic the structure of the basic ECM and allows the material to be fabricated into a variety of shapes, including string-shaped, ribbon-shaped, spiral-shaped, porous, shell-shaped, and tube-shaped [73]. In addition, it has numerous advantages, including a similarity to ECM in its structure, hemostasis, and exudate absorption, which have contributed to its widespread use in biomimetic technologies [74]. Three-dimensional printing (3D printing) is a technology that employs a layer-by-layer technique to connect individual layers of material, typically produced through an additive printing process [75]. This technology has a wide range of applications, including the fabrication of cell sheets that mimic tissue structures or blood vessel structures [76]. Layer-by-layer technology is a manufacturing process that involves the deposition of a charged thin-film active layer through the absorption of nanomaterials or polymer electrolytes with opposite charges [77]. Due to its construction via layer-by-layer assembly, the material exhibits a structure analogous to that of the human skin, comprising the epidermis, dermis, and endothelium [78]. A multitude of biomimetic studies employ these technologies, and the specific applications may vary depending on the methodology employed. Table 1 presents a summary of the findings from these disparate studies. Wound healing is a crucial aspect of wound care techniques as it is a vital response to healing injuries, such as those affecting the skin and organs [79].

Biomimetic wound healing materials such as collagen and gelatin are biocompatible with human skin [80]. Collagen supports tissue regeneration by promoting the migration of fibroblasts and keratinocytes and stimulating ECM production. However, it is expensive and lacks tensile strength in certain wounds [81,82]. It also carries a risk of pathogen transmission due to its animal source. Gelatin is less expensive than collagen, but degrades more rapidly than collagen, requiring more frequent dressing changes. It is not suitable for wounds with heavy exudate [83]. Therefore, their limitations must be considered when choosing wound closure materials. In contrast, biodegradable synthetic polymers including polyglycolic acid (PGA), polylactic acid (PLA), and Poly(ε-caprolactone) (PCL) offer controlled biodegradation rates, good mechanical properties, and minimized dressing changes, in addition to biocompatibility and low immunogenicity [14,84]. These properties can address the limitations of collagen and gelatin.

**Figure 2:**
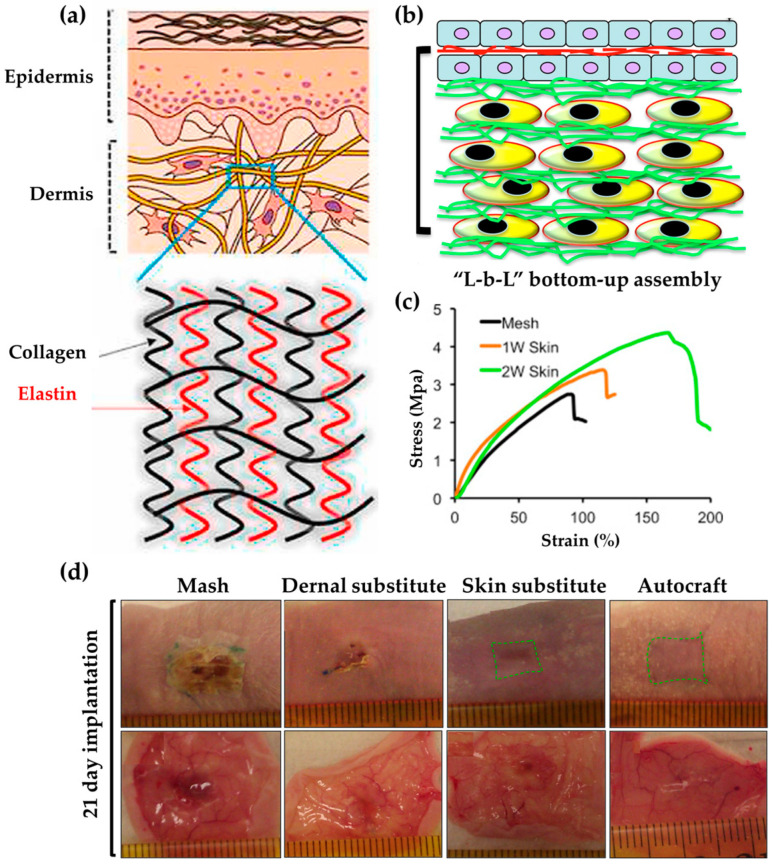
Wound healing mimicking skin. (**a**) Tissue morphology mimicking skin. (**b**) Layer-by-layer (L-b-L) cell assembly structure of nanofiber-based skin substitute. (**c**) Representative stress staining curves of cultured skin substitutes. (**d**) Full-thickness wound healed with graft over 21 days. (**a**): Reproduced with permission from [85], Copyright 2020 Elsevier Ltd. (**b**–**d**): Reproduced with permission from [86], Copyright 2015 Elsevier Ltd.

Medical-grade polycaprolactone (mPCL) was designed to be biomimetic and manufactured using MEM (Melt Electro writing) technology, which created biomimetic structures (Figure 2a) [85]. The resulting mPCL fiber structure was used as a wound dressing with human gingival tissue pluripotent mesenchymal stem/stromal cells. When comparing the regenerative capacity of the dressings over a period of 6 weeks, the researchers confirmed that the dressing could regulate humidity by presenting a moist surface for the first 2 days. Furthermore, the dressing was found to reduce wound contracture and significantly improve regeneration compared to the control group.

Skin substitutes that mimic human skin cells play a role in the treatment and management of burns and wounds. Therefore, a substitute skin based on nanofibers was developed using a cell assembly approach: fibroblasts were fabricated using a layer-by-layer method with fibroblasts on the bottom and keratinocytes on top, along with electro-spun PCL/collagen (Figure 2b) [86]. This mimetic skin substitute exhibited excellent strength with a mechanical strength of up to 4.0 MPa (Figure 2c). Furthermore, when implanted into a full-thickness wound, re-epithelialization occurred, and the wound closed in 21 days. Such biomimetic nanofiber-based cellular assemblies can best treat acute wounds (Figure 2d).

The skin of a fetus in early pregnancy can heal without scarring, even when wounded. To replicate this, the researchers created a wearable biomimetic film (WBMF) composed of hyaluronan, vitamin E, dopamine, and beta-cyclodextrin [87]. The film, which replicated the fetal condition, reduced the inflammatory response by modulating the initial wound situation, resulting in faster wound closure compared to the control (gauze) from day 3 to day 7. The percentage of TNF-α was below 10% and 4%, respectively. It achieved this by inducing fibroblast migration, suppressing the overexpression of transforming growth factors, and aiding in the synthesis and reorganization of collagen. WBMF accelerated wound healing and promoted scar-free wound repair due to normal dermal collagen structure.

**Figure 3:**
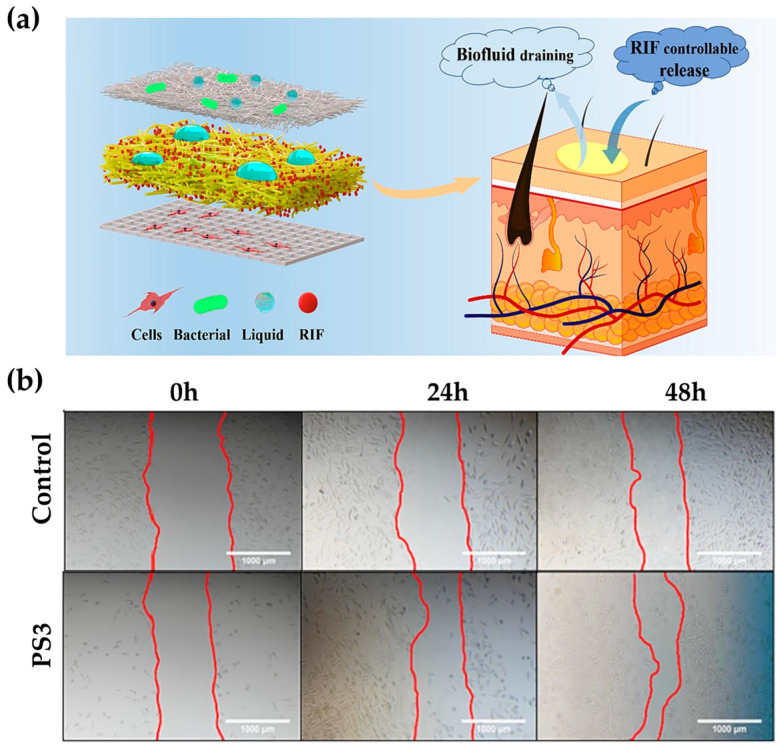
(**a**) Schematic of 3D composite scaffold mimicking skin structure. (**b**) Gap closure in monolayer cells of control and composite scaffolds (PS3) in culture Petri dishes. (**a**,**b**): Reproduced with permission from [88], Copyright 2024 Elsevier B.V.

A 3D composite support, which mimics skin, can promote wound healing [88]. The bottom layer was created using electrohydrodynamic jet printed PCL (BPCL). Lattice-structured aromatic fibers were then printed using a PCL solution to mimic the collagen fibers in the dermis. The subsequent layer utilized rifampicin (RIF) to generate a membrane that maintains an optimal moist wound environment by absorbing tissue exudate. For the final top layer, a hydrophobic PCL membrane (TPCL) was created to mimic the epidermis, the barrier layer to skin wounds (Figure 3a). The 3D composite support mimics the physical, chemical, and mechanical properties of the skin, ensuring proper water absorption, breathability, and hydrophobicity similar to the properties of the epidermis and dermis. In comparison to the control, which had water absorption capacities of 67.5% and 22.9%, respectively, the 3D composite support achieved a water absorption capacity of more than 150%, which falls within the ideal range of 100–900%. In addition, it used pH-responsive micro-patterns to detect exudate changes and release drugs accurately. In vivo assays demonstrate that this approach led to more optimized healing, with cells increasing scratch healing by up to 30% more than controls, and almost all wounds healing in 10 days (Figure 3b).

Wounds are susceptible to secondary infections from external contamination during the healing process. Therefore, antimicrobials are often used to prevent bacterial infection [40]. Inadequate treatment of wound infections can be challenging and potentially dangerous. Symptoms of chronic wounds may include pain or discharge from the wound, fever, and progression of decay [89]. In severe cases, it can lead to systemic infection, sepsis, and multiple organ dysfunction syndrome, which can be life-threatening [27]. Preventing wound infections is crucial to prevent these diseases and promote rapid wound healing.

**Figure 4:**
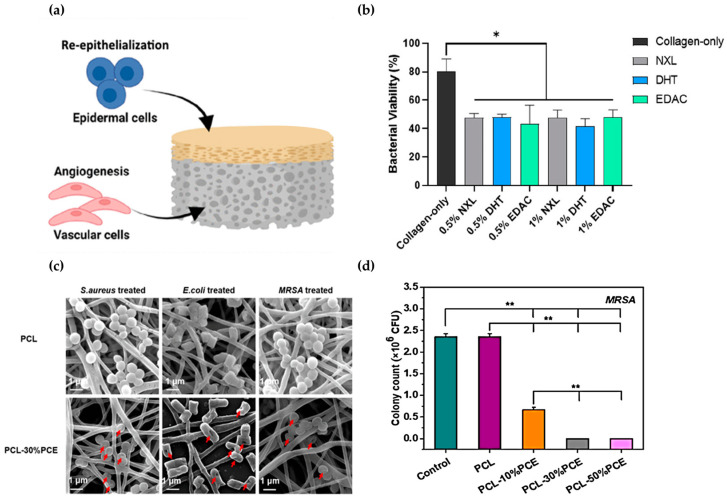
Image of antibacterial mimicking skin. (**a**) Structure of a double-layer antibacterial scaffold mimicking the epidermis and dermis of the skin. (**b**) Viability of *S. aureus* on scaffolds. (**c**) Morphological changes of *S. aureus*, *E. coli*, and MRSA in nanofiber matrix cultures (red arrows indicate morphological changes in the bacterial cell membrane). (**d**) *S. aureus*, *E. coli*, and MRSA colony matrix treated with nanofibers. * *p* < 0.05 and ** *p* < 0.01. (**a**,**b**): Reproduced with permission from [90], Copyright 2023 American Chemical Society (CC BY 4.0). (**c**,**d**): Reproduced with permission from [91], Copyright 2018 American Chemical Society.

Diabetic foot ulcers (DFUs) are a common complication in diabetic patients. These chronic wounds can become open wounds that are at high risk of infection if they do not heal properly. To address this issue, bilayer scaffolds have been developed to mimic the structure of the epidermal and dermal layers of the skin (Figure 4a) [90]. The bilayer scaffolds can be designed to promote angiogenesis and wound healing, which are the functions of endothelial cells. Additionally, to reduce the risk of infection, the epidermis can be fabricated as an antimicrobial barrier layer. The scaffolds that mimic this structure consisted of a porous scaffold layer for the dermis to support angiogenesis and enhance wound healing. Furthermore, an epidermal antimicrobial collagen/chitosan layer was added to prevent infection. The fabricated epidermal layer exhibited a high level of antibacterial activity, resulting in a 60% reduction in bacterial growth when exposed to 1 × 10^6^ *Staphylococcus aureus* (*S. aureus*) (Figure 4b). Additionally, the dermal layer stimulated the growth of keratinocytes and blood vessel cells. The development of a biomimetic bilayer antimicrobial collagen-based scaffold provided a novel approach to improve the healing of DFUs by preventing infection and promoting angiogenesis. The scaffold’s ability to inhibit the growth of *S. aureus* and promote wound healing may have applications in the healing of wounds prone to infection, such as diabetic wounds and pressure ulcers.

The regeneration of tissue is promoted through a mediated cellular microenvironment by structures that biomimic the ECM. To treat diabetic wounds, the researchers developed a nanofibrous scaffold (CPB) that mimics the bone ECM [92]. The nanofiber scaffold was fabricated using ECM components such as collagen, PCL, and bioactive glass nanoparticles (BGNs). Collagen, a mimetic protein, and the nanoparticles, which mimic biological hydroxyapatite, are both ECM components. The CPBs exhibited a greater number of filopodia and a wider spreading area compared to the absence of nanoparticles. High levels of the CD31 protein, which aids in early wound healing by assisting endothelial cells in engrafting new blood vessels, were identified in the CPBs. This was also observed in the CP, but the activated values were higher and more evenly distributed in the CPBs. These scaffolds facilitated the healing of diabetic wounds in vivo, resulting in 60% wound closure in 7 days and 90% in 14 days.

To achieve biomimetic viscoelastic properties, good biocompatibility, and antimicrobial functionality, the researchers developed electro-spun porous nanofibers that mimic the cellular and matrix structures of skin [91]. These nanofibers, composed of poly-ε-poly lysine (PPL) and PCL, were used to combat multidrug-resistant (MDR) bacterial infections. The nanofibers were effective in preventing infections due to their photoprotective and antimicrobial hybrid polypeptide composition. After being exposed to the nanofibers for over two hours, the antibacterial activity against

*Escherichia coli* (*E. coli*), *S. aureus*, *Pseudomonas aeruginosa* (*P. aeruginosa*), *Enterococcus faecalis* (*E. faecalis*), and Methicillin-resistant *Staphylococcus aureus* (MRSA) was over 99.99% (Figure 4d). The bacteria’s morphology indicated that the nanofibers activated an antibacterial mechanism that caused cell membrane disruption (Figure 4c). Additionally, the nanofibers improved skin thickness wounds and skin regeneration, making them an excellent dressing for both antibacterial and regenerative purposes.

**Figure 5:**
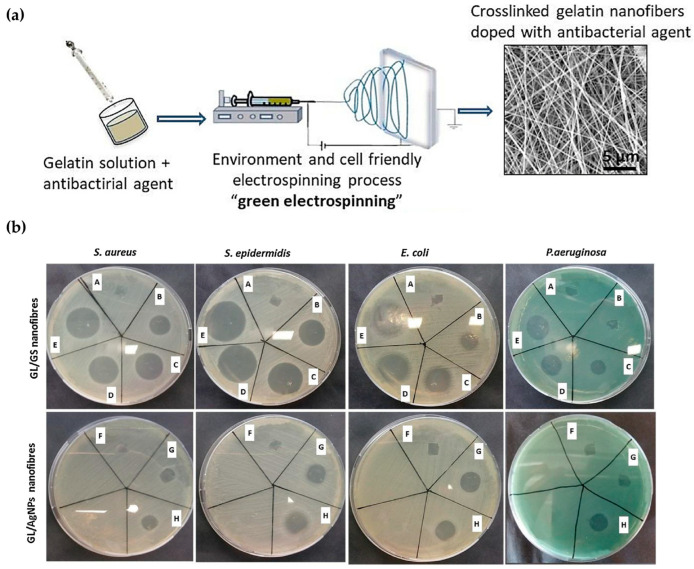
(**a**) Schematic of a nanostructured scaffold with biomimetic and antibacterial properties that mimics ECM. (**b**) Degree of bacterial inhibition by the nanostructure support. (**a**,**b**): (A) drug-free GL, (B) GL/gentamycin(GS)2.5, (C) GL/GS5, (D) GL/GS7.5, (E) GL/GS10, (F) drug-free GL, (G) GL/AgNPs2.5 and (H) GL/AgNPs5. Reproduced with permission from [93], Copyright 2018 Elsevier B.V.

Nanofibers that imitate the ECM can promote normal skin wound healing and exhibit antimicrobial properties. To achieve this, a gelatin (GL) solution containing silver nanoparticles (AgNPs) was prepared and electro-spun into nanofibers after the addition of a (3-glycidyloxypropyl) trimethoxy silane (GPTMS) crosslinker (Figure 5a) [93]. The ECM played a crucial role in preventing bacterial colonization. No bacterial colonization was observed in any of the four bacteria tested, namely *E. coli*, *S. aureus*, *Staphylococcus epidermidis* (*S. epidermidis)*, and *P. aeruginosa*. The method effectively inhibited both Gram-negative and Gram-positive bacteria, with an average inhibition rate of 97% for *S. aureus* and *S. epidermidis*, 98% for *E. coli*, and 93% for *P. aeruginosa* (Figure 5b). Regarding tissue growth, growth was observed on the ECM-like support.

**Table 1:** Summary of cases that mimic skin and in vivo structures.

Application	Inspiration	Material	Function	Ref.
Wound healing	Skin biological structure	mPCL	Skin regeneration, scar reduction, cell therapy	[85]
	Skin cells	PCL, collagen	Promotes mechanical strength, wound healing, suture	[86]
	Fetal skin in early pregnancy	Hyaluronan, vitamin E, dopamine, β-cyclodextrin	Anti-inflammatory, cell migration, collagen synthesis, distribution, reconstruction, scar minimization	[87]
	Skin (endothelium/dermis/epidermis)	PCL, RIF	Antibacterial, moisture retention, penetration gradient, moisture absorption, breathability, hydrophobicity, drug release according to pH changes, wound healing	[88]
	Wound healing of oral mucosa	Chitosan, sodium alginate, EDTA, EGF	Wound healing, scar minimization, sterilization, moist environment, growth factor expression	[94]
	ECM of the dermis	Bovine collagen type I, tropoelastin	Regenerated skin	[95]
	ECM	PCL, cellulose acetate, chitosan, collagen I	Biocompatibility, cell adhesion, spreading, regenerative skin, blood vessel formation, protein upregulation, skin regeneration	[96]
Antibacterial	Skin (epidermis/dermis)	Collagen, chitosan	Angiogenesis, structural stability, antibacterial, cell proliferation, diabetic wound healing	[90]
	ECM	Collagen, PCL, bioactive glass nanoparticles	Diabetic wounds, cell proliferation, angiogenesis, collagen remodeling	[92]
	Skin cells, matrix structure	PPL, PCL	Biocompatibility, wound healing, skin regeneration	[91]
	ECM	AgNPs, gelatin, GPTMS	Wound healing, tissue regeneration	[93]

Abbreviations: AgNPs (silver nanoparticles); ECM (extracellular matrix); EDTA (Ethylenediaminetetraacetic acid); EGF (Epidermal Growth Factor); GPTMS ((3-glycidyloxypropyl) trimethoxy silane); PCL (Polycaprolactone); PPL (poly-ε-poly lysine); RIF (rifampicin).

In the context of contemporary skin biomimetic wound treatment techniques, the objective is to develop functions that facilitate wound healing or healing by emulating the structure or mechanism of the skin. However, this singular objective does not encompass multiple functions within the wound healing process. Consequently, it is unable to respond to complex processes or types of wounds. Therefore, it is essential to integrate various technologies based on biomimetic technology. These include advanced nanofibers, three-dimensional printing, and the loading of smart devices or drugs [97]. The integration of novel technology with skin-mimicking technology necessitates the implementation of a multitude of processes, which will facilitate the customization of treatments for individual patients and the monitoring of their condition.

## 3. Biomimetics of Animal- and Plant-Driven Wound Care Techniques

### 3.1. Wound Care Techniques Inspired by Animals

Animals have been a source of inspiration in the field of biomimicry due to their ability to evolve over millions of years of natural selection in various aspects such as structure, function, environmental adaptation, and information processing [98]. Table 2 provides a synopsis of studies that have employed wound care techniques inspired by these animals.

Lizards have been utilized in numerous biomimetic applications due to their tail autotomy, climbing ability, and adhesion [99,100]. Several lizards use these features to heal wounds.

**Figure 6:**
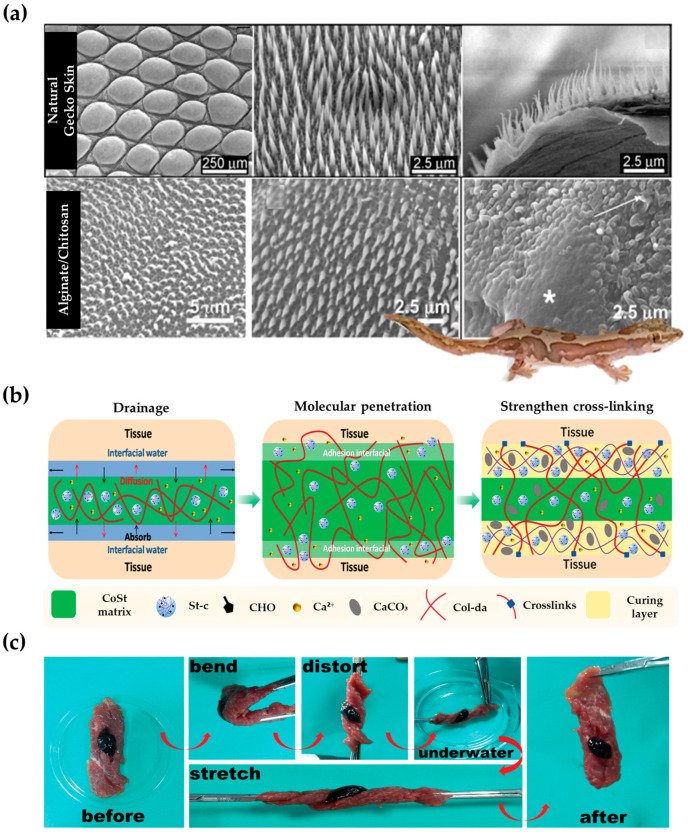
Wound care techniques mimicking animals. (**a**) SEM images of a lizard’s skin surface, spines, and overall view of the skin surface (**top**) and a replica lizard skin with a surface made of a bilayer of chitosan and alginate, spines, and ruptured bacterial debris (**bottom**). (**b**) Tissue adhesion mechanism of CoSt hydrogel and (**c**) shape of the hydrogel when bent or twisted. (**a**): Reproduced with permission from [102], Copyright 2017 Springer Nature. (**b**,**c**): Reproduced with permission from [103], Copyright 2022 Wiley-VCH GmbH.

Barn lizards possess nanotip hairs that cover their skin and have antibacterial properties, which kill bacteria (Figure 6a) [101]. To replicate this effect, a synthetic lizard skin surface was created using polystyrene (PS) and several biopolymers [102]. The surface was able to destroy over 95% of bacteria by rupturing their cell membranes, similar to the way a lizard’s skin repels bacteria (Figure 6a).

The Texas horned lizard’s skin has a network of microstructures between its scales that form a specialized capillary system. This network allows for predetermined directional fluid flow, giving the liquid mobility. To replicate this system, the researchers developed a microfluidic device that collects and transports wound exudate, allowing for precise biomarker detection at the point of care [104]. As wound fluid developed, it was directed through a microfluidic layer to the sensing area via serrated capillaries that promote continuous flow in one direction. This liquid transport system captured 180% of wound fluid and delivered it to the sensor in less than 130 s, demonstrating the reliability of the fabricated system. The exudate is collected and then analyzed using a wireless smartphone-based data readout. This enabled timely and personalized treatment to assist with wound healing.

Mussels are marine organisms that possess adhesive properties and belong to the category of marine adhesive animals. The adhesive proteins and genes of mussels enable them to attach to various surfaces in the water [105]. Because of these characteristics, biomimicry has become widely used in the field. Adhesion does not require an additional fixation process using a secondary bandage on the wound. The hemostatic effect and strong adhesion prevent tissue exudate absorption and bacterial infection, greatly aiding with wound healing [106,107,108].

Mussels attach to underwater surfaces using catechol side chain groups from attachment foot proteins. They connect with surfaces using a variety of bonds, such as π-π, hydrogen, and electrostatic interactions. The researchers developed a highly adhesive collagen–starch-based (CoSt) hydrogel with excellent wound-healing properties inspired by these adhesion methods and geometries (Figure 6b) [103]. To mimic the mussel’s adhesive system, dopamine groups were introduced into collagen chains (Col-da). Furthermore, the aldehyde group of the chain (St-c) was modified to mimic the uronic acid residue, as starch has a sequence and structure similar to AGP, one of the mussel adhesive protein components. The collagen and starch formed a network through covalent and non-covalent bonds under calcium ions, resulting in a hydrogel that mimics the mussels’ attachment system. The results indicate that the tested material exhibited high adhesion and sealing strength, with an adhesion strength of 60 kPa and a sealing strength of 150 mmHg (Figure 6c). This property allowed for rapid hemostasis, confirmed in various parts of the mouse, including the tail and liver, with a time of 20 to 25 s. This excellent hemostatic ability promoted the skin’s regenerative capacity, allowing for the complete healing of incision wounds in just 10 days.

**Figure 7:**
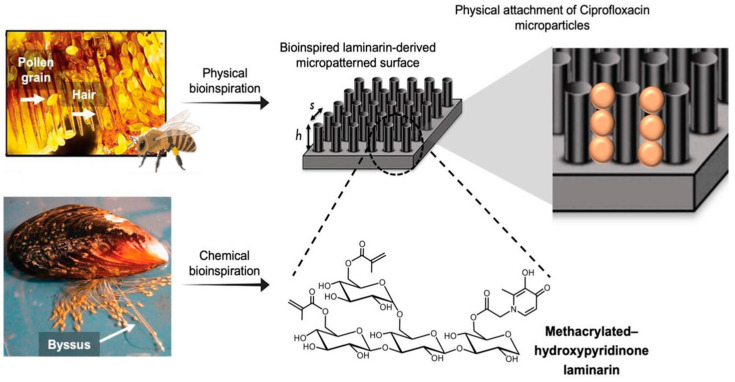
Schematic diagram of inspired mussel and bee hair crevice patches. Reproduced with permission from [109], Copyright 2023 Wiley-VCH GmbH.

A bee’s leg hair contains millions of pollen grains and particles due to optimized physical adhesion between the hair and pollen (Figure 7). Inspired by this, the researchers created micropatterned polydimethylsiloxane (PDMS) structures that efficiently trap large amounts of microparticles [109]. They combined the adhesive power of natural mussels to produce a naturally derived pharmaceutical package with bio-adhesive properties. The patch was made using laminarin (LAM), which promoted wound healing, and was applied to a fine pattern patch. By modifying LAM with a meth acryloyl group (LAM-MET), it was possible to manufacture a hydrogel. However, its use had been limited due to its slightly adhesive strength. Inspired by the strong adhesiveness of mussel byssus to the outer shell, the researchers created a patch by introducing hydroxypyridinone (HOPO), a catechol-like molecule, into the polymer skeleton. It has been confirmed that the introduction of HOPO improved the level of adhesion by approximately 3.89 times to 1.4 kPa compared to the patch without introduction. Additionally, for fine particles, the drug’s adhesion efficiency was increased. However, for non-micropatterned particles, the capture efficiency was only 19.3% compared to the micropatterned hydrogel. These patches were designed based on natural phenomena and the various functions of LAM that promote wound healing. This resulted in high adhesion and increased drug capture efficiency.

### 3.2. Wound Care Techniques Inspired by Plants

Plants have evolved over 400 million years and serve various functions, such as providing mechanical support, reflecting and absorbing light, regulating moisture, and exhibiting adhesive and non-adhesive properties [110]. Biomimetic research and application of functions of natural systems in different areas [111].

**Figure 8:**
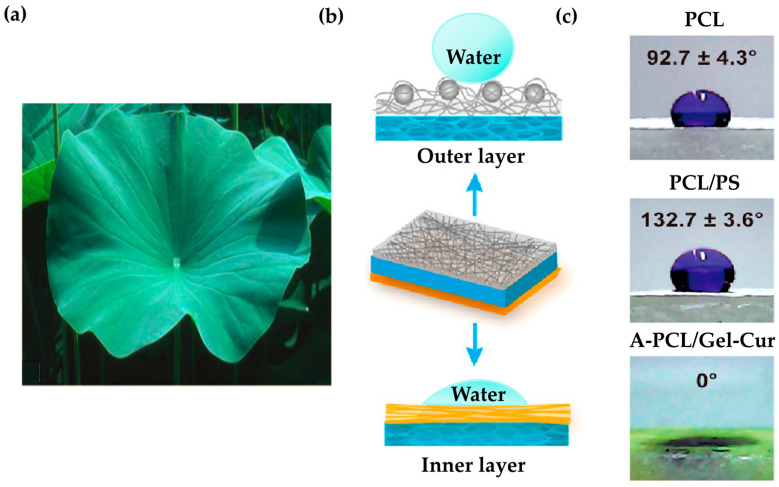
Wound management technology mimicking plants. (**a**) Lotus leaf on hydrophobic surface. (**b**) Schematic diagram of the asymmetric composite dressing and (**c**) contact angle image of the dressing layer (right) (**a**): Reproduced with permission from [112] Copyright 2011 Beilstein-Institut (CC BY 4.0). (**b**,**c**): Reproduced with permission from [113], Copyright 2022 American Chemical Society.

The lotus leaf’s surface structure is superhydrophobic, which increases the material’s roughness and reduces its contact area with water (Figure 8a) [112]. The researchers developed a micro nanostructure that mimics this superhydrophobic surface, significantly improving the material’s hydrophobicity (Figure 8b) [113]. This enabled the fabrication of asymmetric dressings with high hydrophobicity and antibacterial adhesion to the outer layer. The material prevented the accumulation of exudate and excessive wound hydration, while also exhibiting antibacterial properties. To replicate the surface structure of a lotus leaf, PCL was combined with PS microspheres and fabricated using electrospinning. The resulting micro nanostructure surface closely resembled the size and structure of lotus leaf protrusions, successfully imitating its surface structure. It has been confirmed that water droplets did not penetrate the middle layer of the sponge when they encountered its surface, but instead remained on the surface. The contact angle of the mimicked surface was 40.1° greater than that of the pure surface, confirming its excellent hydrophobic properties (Figure 8c).

**Table 2:** Summary of cases of imitating plants and animals.

Inspired Organism	Inspired Species	Inspiration	Application	Material	Function	Ref.
Animal	lizard	Small nano-tipped hairs (thorns)	Antibacterial	Chitosan, alginate, silk fibroin	Antibacterial, hydrophobicity	[102]
		Directional fluid flow skin	Sensing	Graphene, gold, graphite	Sensing, exudate collection	[104]
	mussel	Adhesion	Hemostasis, wound healing	Collagen, starch	Adhesion, sealing, hemostasis, healing	[103]
		Adhesion	Drug release	Methacrylated laminarin, hydrogel	Adhesion, drug content, drug release, antibacterial	[109]
	native blood vessels	plant cellulose-based blood vessel	Artificial blood vessels	Cellulose hydrogel, HEMA	Mechanical performance, cytocompatibility, hemocompatibility, artificial blood vessels	[114]
Plant	lotus leaf	Skin structure, superhydrophobic structure	Wound healing	Collagen, chitosan, PCL, PS, Curcumin, gelatin	Antibacterial, healing, cell growth, hydrophobicity	[113]
		fine structure of leaves	Sensing	PDMS, PPy/Ag hybrid	Electrical conductivity, pressure, stretch and bend sensors	[115]
	Delosperma cooperi	Centripetal multilayer structure	Self-sealing	PDMS, shape memory polymer	Self-sealing	[116]

Abbreviations: HEMA (hydroxyethyl methacrylate); PCL (Polycaprolactone); PDMS (Polydimethylsiloxane); PPy (Polypyrrole); PS (Polystyrene).

## 4. Biomimetic Nanoparticles for Wound Care Techniques

### 4.1. Wound Care Techniques Inspired by Nanostructure

Nanomaterials and substances have been applied in biomimetics due to their similar structures and sizes to molecules, providing various approaches to cell or tissue compartments [117,118]. To utilize this opportunity, numerous studies are conducting nano biomimetic research inspired by the properties, structures, and applications of nanomaterials. Examples of such research include nanovesicles, nanozymes, nanostructured biosilica, and nanosynthetic systems, which are summarized in Table 3 [119,120].

Nanovesicles are nano-sized structures composed of various types of nanoparticles. Due to their fine structure and ability to absorb or trap various substances, they have garnered attention for their potential applications in various fields of life and medicine. They have also been utilized as an important material in the field of biomimetics [121].

**Figure 9:**
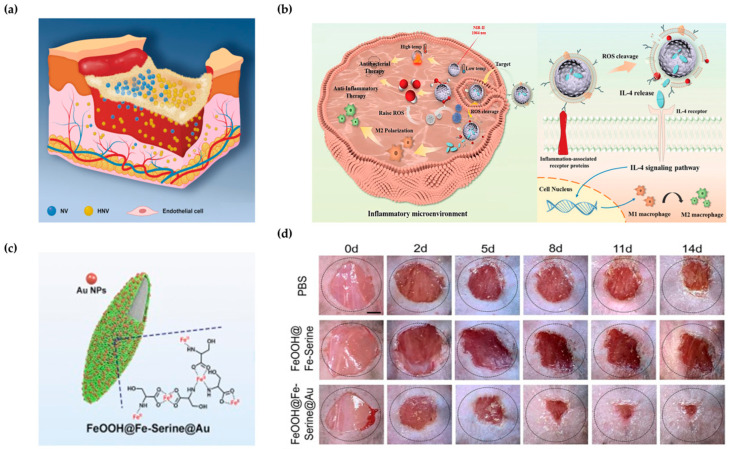
Wound management technology mimicking nanostructures. (**a**) Schematic diagram of biomimetic nanovesicles. (**b**) Bacterial biofilm and anti-inflammatory therapy of biomimetic nanozyme and IL-4 release mechanism. (**c**) Schematic diagram of the biomimetic dual nanozyme and (**d**) regeneration in diabetic infected wounds. (**a**): Reproduced with permission from [122], Copyright 2023 Elsevier B.V. (**b**): Reproduced with permission from [123], Copyright 2023 Wiley-VCH GmbH. (**c**,**d**): Reproduced with permission from [124], Copyright 2023 Elsevier Inc.

Endothelial cell-derived nanovesicles (NVs) promote angiogenesis. However, their effectiveness in diabetic wounds is limited due to oxidative stress and imbalanced cell proliferation during infection. To address this issue, biomimetic hybrid nanovesicles (HNVs) were used [122]. HNVs have improved drug loading efficiency and were produced by hybridizing rhamnolipid liposomes with NVs (Figure 9a). The in vivo penetration rate of NVs was 49.04%, compared to HNVs through biomimetics. When confirmed by penetration analysis, it was found that HNV penetrated deeper than NV. This suggests that HNV may have a better ability to penetrate within the complex crevices caused by diabetic wounds. As a result, even in actual infected diabetic wounds in vivo, HNV healed the wound in 14 days, whereas the control group healed slowly, and exudate was visible. According to the text, HNV, a biomimetic hybrid nanovesicle, has better antibacterial activity, which promoted targeting of endothelial cells in infected diabetic wounds.

Nanozyme is an artificially designed nanomaterial with enzyme-like properties that combines the features of chemical catalysts and biocatalysts. It is used in various fields, including antibacterial and tumor applications. Due to its enzyme-like activity and involvement in various in vivo reactions, it has been widely used in biomimicry [125].

In the field of biotherapeutics, single-atom nanozymes (SANs) generate reactive oxygen species (ROS) through photothermal and glutathione depletion functions, effectively hindering biofilm growth. However, despite these approaches, the formation of microbial biofilms can lead to chronic inflammation. Further research is needed to control it. The synthesis method of the nanozyme can be controlled to introduce appropriate immunogenic building blocks for fabricating biomimetic single-atom immunomodulatory nanozymes.

Synthetic nanoparticles (NPs) can be encapsulated by immune cell membranes, such as neutrophil membranes and macrophages, and targeted to sites of inflammation during blood circulation. Thus, the researchers developed a hollow mesoporous biomimetic single-atom nanozyme (SAN) that mimics the homing mechanism of natural macrophages [123]. This nanozyme targeted the site of inflammation, inhibited factors, and eliminated bacteria to enhance the disruption of biofilm growth (Figure 9b). The biomimetic nanozyme (Co@SAHSs@IL-4@RCM) comprised hollow spheres embedded with cobalt SAN, which was loaded to encapsulate IL-4 into the Raw 264.7 cell membrane. When the biomimetic nanozyme accumulated in the infected area through cell membrane receptors, it penetrated and destroyed the biofilm structure. This process induces the release of IL-4, which can reorganize macrophages, inhibiting oxidative damage and tissue inflammation (Figure 9b).

AuNPs mimic the glucose oxidase enzyme (Gox), which catalyzes glucose into gluconic acid and H_2_O_2_. Accordingly, a Gox- and peroxidase (POD)-mimicking dual nanozyme catalytic cascade system was constructed by partially reducing Fe^3+^ to Fe^2+^ and depositing AuNPs (Figure 9c) [124]. This mimicking system secured the use of Gox in diabetic wound infections, which has many limitations due to complex preparation, cost issues, and sensitivity to the environment. The Gox mimetic environment utilized gold to convert glucose and oxygen into gluconic acid and hydrogen peroxide. The POD-mimicking environment employed iron ions from hydrogen peroxide to generate hydroxyl radicals and water. This chain catalytic mimetic enzyme activity depletes glucose and acidifies the wound environment, inhibiting bacterial growth and providing adequate hydrogen peroxide in an alkaline environment. In vivo, these biomimetic dual nanozymes almost completely healed diabetic wounds infected, unlike the control group, where chronic wound ulcers were observed (Figure 9d). Additionally, it demonstrated promising therapeutic efficacy against diabetic wound infections with an antibacterial efficacy of up to 99.1%.

### 4.2. Wound Care Techniques Inspired by Diatom and Diatomite

Diatoms are a type of single-celled algae that can range in size from 2 μm to 2 mm. Diatomite, which is a deposit of their shells, is a popular natural source of nanostructured silica [126,127]. This process results in the creation of a frustule, which is a siliceous micro- to nano-porous cell wall. The frustule has a complex pore pattern, which leads to features such as high surface area, porosity, and biocompatibility [128]. This material is commonly used in various industrial applications due to its advantages and low cost [129,130]. Research has been conducted on wound care technologies inspired by diatoms and biomimetic technologies using diatomite due to their advantages [131]. Adhesives play a crucial role in wound care techniques as they are utilized for wound closure and hemostasis [108,132].

**Figure 10:**
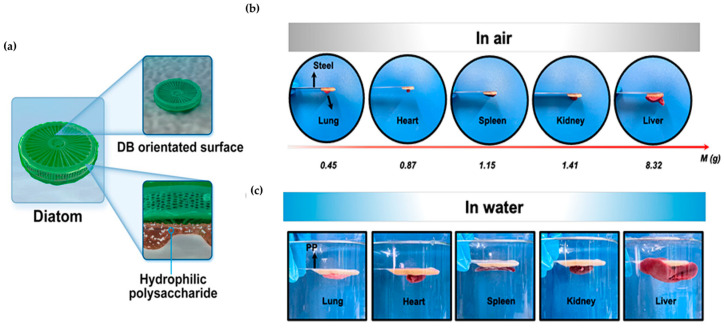
Diatom-mimicking wound care technology. (**a**) Schematic of a diatom-mimicking polysaccharide adhesive and its adhesive properties (**b**) in air and (**c**) in water with biological tissues. (**a**–**c**): Reproduced with permission from [133], Copyright 2023 American Chemical Society.

In aquatic environments, diatoms secrete hydrophilic extracellular polymeric substances that enable them to adhere to aquatic surfaces. To replicate this process, the researchers utilized *Bletilla striata* polysaccharide (BSP), a naturally occurring polysaccharide, to provide its structural composition and biocompatibility (Figure 10a) [133]. The replicated BSP was combined with diatom biosilica (DB) and utilized as an occlusive hemostatic agent. The hemostatic agent was confirmed to adhere to various biological organs and objects weighing 0.45 to 25.25 g (Figure 10b,c). It achieved rapid hemostasis within 1 min due to its excellent coagulation ability.

A biomimetic universal adhesive was created through the radical polymerization of tetrahydrofurfuryl methacrylate (THMA) and methyl acrylate (MA). Inspired by aquatic diatoms, this adhesive demonstrated stable adhesion [134]. Notably, its adhesive strength on pig casing and titanium alloy substrate remained over 150 kPa even after 50 h, indicating its potential for long-term use. Furthermore, the hemostatic ability of the bleeding area was verified as it prevented 160 mg of blood loss from the injured liver of the mouse, in contrast to the case without adhesive.

Diatomite is a natural mineral substance formed by the deposition of silica-containing carcasses in diatom cells. It contains polar silanol groups on its surface, which can effectively promote blood coagulation [135]. The researchers used this structure to adsorb silver ions onto yeast to simulate diatomaceous earth cells and wrapped mesoporous silicon around the yeast to create silver-coated porous hollow silica spheres [136]. The resulting complex significantly improved blood hemostasis efficiency, reducing blood coagulation time by 290 s compared to the blank control group. Furthermore, the AgNPs added to the complex exhibited a 70% antibacterial rate against E. coli, confirming the complex’s ability to achieve hemostasis, control bacterial infection, and promote wound healing, inspired by the structure of diatomite.

The diatom structure consisted of microscopic pieces of silica known as diatomite. To address the limitations of a two-dimensional platform for loading antibacterial ingredients in nanomaterials, the researchers developed a nano-protrusion plate inspired by this structure [137]. Mesoporous silica layers cover the top and bottom surfaces of oxidized graphene oxide sheets using tetraethyl orthosilicate (TEOS) and mercaptopropyl trimethoxy silane (MPTMS), which are silica-related compounds. A nano frustule loaded with nitric oxide through S-nitration was created, with an average pore size of 3 nm, resulting in a diatomite-like pore structure. The nano frustule exhibited strong and non-specific antibacterial activity against S. aureus and E. coli, with a half minimum inhibitory concentration (MIC_50_) of 250 μg ml^−1^. The mechanism of action involves killing bacteria in 2D porous structures by exploiting entropy-driven aggregation behavior.

**Table 3:** Summary of cases of mimicking NPs.

Inspired Organism	Inspired Species	Inspiration	Application	Material	Function	Ref.
Nanostructure	Nanovesicles	Endothelial cell-derived nanovesicles	Infection diabetic wound	Extruded endothelium, rhamnolipid liposome	Antibacterial, targeting, penetration	[122]
	Nanozyme	Homing mechanism of natural macrophages	Wound healing	RAW 264.7, IL-4, Cobalt	Targeting, anti-inflammatory, antibacterial, antioxidant, immunomodulatory	[123]
		Glucose oxidase, peroxidase	Infection diabetic wound	Fe^2+^, AuNPs	Antibacterial, healing	[124]
Diatomaceous	Diatom	Adhesion	Hemostasis	DB, BSP	Adhesion, hemostasis	[133]
		Adhesion	Adhesion	THMA, MA	Adhesion	[134]
	Diatomite	Structure	Antibacterial, hemostasis	Silver, hollow polymerized silicon NPs	Antibacterial, hemostasis	[136]
		Siliceous frustule	Antibacterial	Graphen oxide, TEOS, MPTMS	Antibacterial, controlled release	[137]

Abbreviations: AuNPs (gold nanoparticles); BSP (*Bletilla striata* polysaccharide); DB (diatom biosilica); MA (methyl acrylate); MPTMS (mercaptopropyl trimethoxy silane); TEOS (tetraethyl orthosilicate); THMA (tetrahydrofurfuryl methacrylate).

## 5. Electronic Skin (E-Skin)

### 5.1. Concept of E-Skin and Its Multifunctional Capabilities

**Figure 11:**
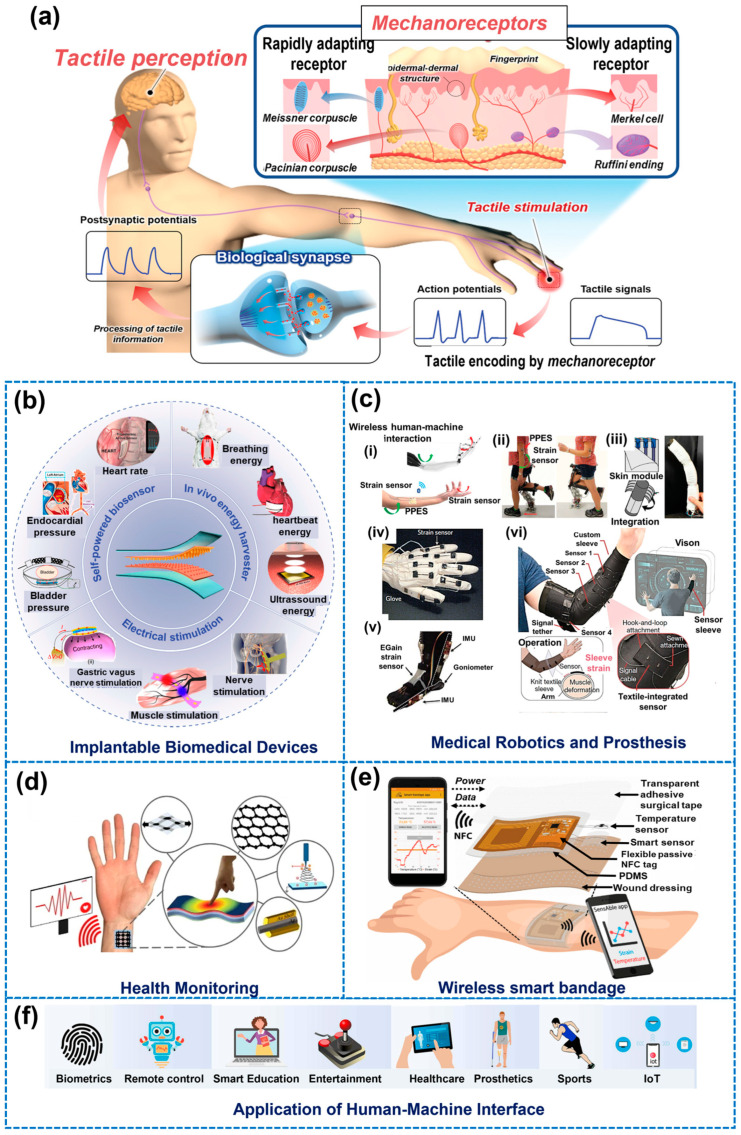
(**a**) Human tactile perception system. (**b**–**f**) Various applications of E-skin. (**b**) Overview of an implantable biomedical device based on Triboelectric Nanogenerators (TENGs). (**c**) Applications of flexible strain sensors in medical robotics and prosthetics. (**d**) Real-time bio- and health signal monitoring. (**e**) Overall design of the wireless smart bandage for chronic wound management. (**f**) Application of the human–machine interface (**a**): Reproduced with permission from [159], Copyright 2019 Wiley-VCH Verlag GmbH & Co. KGaA, Weinheim. (**b**): Reproduced with permission from [160] (CC BY 4.0). (**c**): Reproduced with permission from [161], Copyright 2022 John Wiley & Sons. (**d**): Reproduced with permission from [153], (CC BY 4.0). (**e**): Reproduced with permission from [162], (CC BY 4.0). (**f**): Reproduced with permission from [163], Copyright 2022 OAE Publishing Inc. (OAE) (CC BY 4.0).

The skin, our body’s largest and heaviest organ, serves crucial functions such as protection and sensation. It is estimated that the skin contains around 45,000 mechanoreceptors of four distinct types: Meissner corpuscles, Pacinian corpuscles, Merkel cells, and Ruffini endings [138,139]. Additionally, approximately 17,000 myelinated tactile fibers innervate the surface of the palm [140,141]. The skin also houses thermoreceptors [142] for detecting temperature changes and nociceptors [143] for recognizing pain. These specialized receptors play a vital role in transmitting information about touch, pressure, vibration, and skin tension to the central nervous system (Figure 11a). Researchers have drawn inspiration from the skin’s superior perception capabilities to develop E-skin capable of sensing various physical stimuli with high sensitivity and differentiating them with good temporal and spatial resolutions [139,144,145]. E-skin is an ultra-thin, flexible, and comfortable sensing system that tracks vital physiological data with high sensitivity [146]. By combining sensors and circuits on flexible substrates, this sensing system achieves unique ductility and heightened sensitivity to a wide range of signals [145,147]. Sensing technologies that mimic human skin have garnered significant attention for applications in artificial intelligence [59,148], human–machine interfaces [57,149,150], prosthetics [60,151], health monitoring [59,152,153], soft robotics [61,154,155], disease detection [148,156,157], and health care [158]. Its potential applications are shown in Figure 11b–f.

Wearable electronics and intelligent patches designed to mimic human skin’s functions allow for continuous and non-invasive monitoring of physiological and biochemical information around wounds [68,164,165]. Real-time monitoring can significantly enhance wound healing by enabling early detection and continuous assessment of wounds, leading to better management of patients with chronic illnesses [58,63,65,66,166,167,168,169]. Remote wound management systems through smartphone applications empower patients and caregivers to conduct regular wound scans at home, facilitating early changes in wound management and enhancing patient satisfaction [170]. These technological advancements in electronic capabilities offer promise for improved wound management, early complication detection, and personalized healthcare in wound healing.

### 5.2. E-skin Integration with Wound Monitoring and Management

Wound dressings with these E-skins can monitor wound conditions in real-time to provide timely feedback and treatment [64,67]. They can incorporate intelligent sensing technology to track wound conditions such as pH, infection, and bacterial presence, enabling targeted and personalized medication release [171].

**Figure 12:**
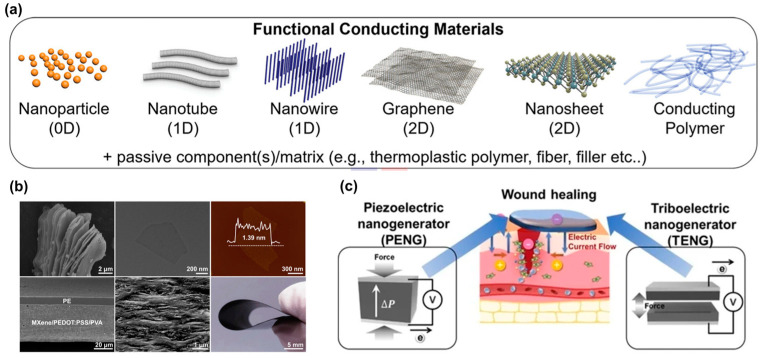
(**a**) Schematic of the materials and structures of conducting materials. (**b**) SEM photos of MXenes: accordion-like unexfoliated MXene, MXene nanosheet, and intercalated MXene nanocomposite film and optical photo of the flexible intercalated MXene nanocomposite film. (**c**) Schematic representation of a piezoelectric and triboelectric nanogenerator on operating on injured skin. (**a**): Reproduced with permission from [175], Copyright 2019 Wiley-VCH Verlag GmbH & Co. KGaA, Weinheim. (**b**): Reprinted with permission from [177], Copyright 2022 Elsevier Inc. (**c**): Reproduced with permission from [79] (CC BY 4.0).

Materials that are mechanically flexible, wireless, and adhere well to the skin have been utilized to monitor and treat wounds [69] (Figure 12). Silver nanowires (Ag NWs) [172], graphene [173], carbon nanotubes (CNTs)/nanofibers (CNFs) [174], conductive polymers [175] (Figure 12a), Ti_3_C_2_Tx (MXene) (Figure 12b) [176,177], and transistors that convert mechanical signals into electrical signals, such as triboelectric nanogenerators (TENGs) [178] and piezoelectric nanogenerators (PENGs) (Figure 12c) [179], have been used as E-skin materials. Electrical stimulation plays a role in accelerating wound healing by improving blood vessel permeability, leading to facilitating the movement of growth factors and immune cells crucial for the healing process and enhancing blood flow and nutrient delivery to the wound site [180]. The use of electrically conductive biomaterials is gaining interest in the E-skin field due to their sensing capabilities through electron transfer, as well as their capacity to promote wound healing through electrical stimulation [181]. Table 4 summarizes some published studies reviewed, including the ingredients used to make the E-skin, its performance, and the monitoring and treatment effects.

#### 5.2.1. Skin-Inspired Antibacterial E-Skin

Researchers have developed skin-inspired, antibacterial conductive hydrogels for epidermal sensors and diabetic foot wound dressings [182,183]. These hydrogels integrate a conductive polymer for electronic components, an antibacterial agent for protection against infections, and mechanical properties optimized for comfort and flexibility resembling human skin. Additionally, a breathable, biodegradable, antimicrobial, and self-powered E-skin made of all-nanofiber TENGs has been developed for real-time monitoring of physiological signals and body motions [184]. This E-skin is fabricated by sandwiching antimicrobial silver nanowire (AgNw) between biodegradable PLGA and PVA. The E-skin offers a micro-to-nano hierarchical porous structure with a large specific surface area for contact electrification and capillary channels for temperature and moisture transmission, enabling real-time monitoring of physiological signals and body movements.

#### 5.2.2. Adhesion and Visualization Techniques Inspired by Nature

In order to develop effective skin-adherent bioelectronics for continuous monitoring of biological and physical signals, researchers have drawn inspiration from natural adhesion mechanisms seen in gecko’s toes and mussels [185,186]. Zhang et al. developed gecko’s feet-inspired self-peeling switchable dry/wet adhesive (SPSA). SPSA was created by combining thermal-responsive hydrogel layers with mushroom-structured arrays inspired by a gecko’s foot and copolymer adhesive coatings inspired by mussels [187]. Another self-healing adhesive hydrogel using mussel-inspired dopamine [188], tea phenols [189], or tannic acid [190] acted as an epidermal or strain sensor. Biomimetic technologies inspired by chameleons, butterflies, and peacocks have led to the creation of E-skins capable of visualizing external stimuli through color changes, providing human-readable output signals for intuitive recognition of stimuli [191]. The periodic arrangement of the complex photonic micro/nanostructures affects the diffraction and reflection of light, resulting in the fascinating color changes observed in these creatures [192,193]. Inspired by the properties of chameleon skin, Ziai et al. embedded silver nanocubes as a photonic device into a poly(N-isopropylacrylamide)-based hydrogel network to create a nanostructured system with enhanced thermo-responsiveness and antimicrobial properties, and used it for glucose sensing [194]. A micropillar array covered in polydopamine (PDA) to improve adhesion was inspired by geckos and mussels [138]. In addition, elastic copolymer NPs of P(MMA-BA) were assembled into a photonic crystal pattern to give the E-skin the ability to detect multiple targets simultaneously, such as biochemical sensing, tactile sensing, motion monitoring, and wound healing.

A novel acellular dermal matrix-based bioelectronic skin (e-ADM) has been manufactured through a one-pot biocompositing strategy with a cicada-wing-like rough surface and nanocone microstructure for wound healing applications [166]. The e-ADM exhibits properties such as high tensile strength, flexibility, biodegradability, electroactivity, biocompatibility, pH-responsiveness, and antibacterial activity. The cicada wings, like the rough surface and nanocone microstructure, enhance the adhesion of functional building blocks, contribute to the mechanical properties of the E-skin, including robust tensile strength and flexibility, and enable the E-skin to exhibit antibacterial properties [195]. These new flexible E-skin sensors can be utilized to accurately monitor the amplitude of various human activities in real time to record injuries and movements. In addition, e-ADM can effectively promote cell growth and proliferation by electric stimulation.

#### 5.2.3. Electrospun Nanofibers for E-Skin Dressings

Resemblance to ECM, increased surface-to-volume ratio, and porousness make electrospun nanofibers an ideal material for E-skin dressings [196,197,198,199]. Self-responsive electrospun nanofibrous wound dressings have been developed using various smart materials or polymers to encapsulate medications and release them based on responses to wound conditions like infection, oxygen species, pH changes, or temperature [196,200]. These dressings enable early detection of complications and improved wound management. For instance, an electrospun composite micro/nanofiber-based bilayer-dressing patch demonstrated a multifunctional dressing patch with healing, protection, and monitoring for advanced wound care [197]. Both the healing-support layer composed of hyaluronic acid, gelatin, and dexpanthenol and the protective/monitoring layer made of curcumin and PCL were electrospun to form micro/nanofibers. This bilayer-dressing patch showed fibroblast growth promotion and biocompatibility, antimicrobial properties, and pH-responsive color changes. The wounds on BALB/c mice were diagnosed by observing the color change of the dressing patch, and the wounds healed rapidly without scarring. An E-skin made of a CNTs/graphene/Gelatin methacryloyl (GelMA) mat can function as a strain sensor, with gauge factors matching the mechanical performance of human skin, making it suitable for monitoring the strain on the skin [201]. The mat functions as moisture sensing, with a sensitivity coefficient indicating its ability to monitor and warn against interstitial fluid outflow from wounds. The integration of the CNTs/graphene/GelMA mat with a portable device enables the monitoring of strain and moisture on a rat model with abdominal wounds, showcasing its potential for wound management and home medical diagnosis.

#### 5.2.4. Biofuel Cell Patch

Researchers have introduced innovative solutions such as self-powered diabetic wound healing patches that utilize high blood glucose to produce electrical energy and ROS for sterilization [202]. A glucose biofuel cell (GBFC) patch can overcome hyperglycemia and low H_2_O_2_ limitation in diabetic wounds. In addition, the electric field generated by GBFCs induces negatively charged bacteria to adhere tightly to the electrode surface, allowing the ROS generated by the electrode to localize to the bacteria, enabling precise sterilization without the need for antibiotics, overcoming the limitations of antimicrobial resistance. Similarly, a smart COF nanoreactor has been developed to induce the in situ generation of hydroxyl radicals for sterilization in diabetic wounds, improving the wound microenvironment [203]. These demonstrate the potential to address challenges in wound care, such as antimicrobial resistance and hyperglycemia, through novel therapeutic approaches.

#### 5.2.5. Wound Monitoring and Treatment System

Wearable bioelectronic systems have been introduced for continuous monitoring of biomarkers, including temperature, pH, ammonium, glucose, lactate, and uric acid related to wound healing processes [204]. These systems combine treatment modalities like drug release from electroactive hydrogel layers controlled by voltage-modulated electrodes with electrical stimulation for tissue regeneration. The combination of controlled anti-inflammatory and antimicrobial therapy with tissue regeneration by electrical stimulation contributed to accelerating the healing process. In the realm of wound dressings, a stretchable, flexible, and breathable bandage has been designed with multiple layers for effective wound monitoring and treatment [205]. By incorporating kirigami techniques and an F-sensor for pH monitoring, this bandage can stretch significantly and provide targeted photothermal therapy (PTT) through the MXene top layer for bacterial inhibition, accelerating the healing process. The combination of pH monitoring and PTT helps inhibit bacterial growth and promotes faster wound healing compared to traditional dressings.

Additionally, an integrated drug delivery and sensing module for wound dressings has been developed, enabling the delivery of growth factors and real-time colorimetric monitoring of glucose levels and pH to promote wound healing and detect infections early on [167]. Furthermore, low-cost, flexible platforms for continuous oxygen delivery and sensing in wound dressings have been introduced, utilizing paper substrates and inkjet printing technology [206]. These platforms aim to mitigate wound hypoxia by increasing oxygen concentration in the wound bed and monitoring oxygen levels effectively.

A smart, thread-based wound dressing that integrates a pH-based early detection system and a topical, on-demand drug delivery system that can deliver antibiotics or other therapeutic agents directly to the wound site has been presented [207]. A multifunctional bilayer hydrogel based on tannic acid and carboxymethyl chitosan responds to the acidic environment of a wound, with the inner layer degrading to release substances that kill bacteria and the outer layer changing color depending on pH, allowing physicians to see how the wound is healing [208]. Zhang et al. developed a soft wound infection monitoring system with pH and temperature sensors to monitor pH and temperature changes early on in the infection in a murine model of infection [209]. Based on a natural porcine skin matrix, the multifunctional E-skin composed of MXene nanosheets, silver nanowires, and mesoporous hollow silica microspheres monitors temperature, pH, and electrical reactivity in real time after melanoma surgery to release anticancer drugs embedded in mesoporous hollow silica microspheres in response to multiple stimuli and promote regeneration with electrical stimulation [210]. Liu et al. developed smart textiles simultaneously, satisfying both thermotherapy and real-time motion monitoring [211]. Carbon black-coated nylon, silver-plated nylon, and elastic spandex yarns, which exhibited flexibility and heating performance, provided hyperthermia to injured joints in response to joint motion measured in a spiral twist structure. An integrated bilayer microneedles dressing and TENGs offer an efficient therapeutic platform for wound management [212]. One layer serves as an electrochemical sensor, while the other is antibiotic-coated for drug delivery. This platform demonstrates antibacterial effects, promotes cell proliferation and migration through electrical stimulation, and monitors wound health by sensing dual markers via redox reactions.

#### 5.2.6. Wireless Smart Dressings with Closed-Loop Systems

Wireless smart dressings with closed-loop sensing and stimulation circuits have also been developed to manage wound exudate, monitor wound conditions, and promote faster healing through synergistic approaches [69,213]. The dressing, developed by Ge et al., can pump and store wound exudate into microfluidic channels, detect wound conditions through temperature and humidity sensors, and control drug release from the PNIPAM-co-AM hydrogel using a smartphone [213]. In mouse models, the combination of wound exudate management and drug release worked synergistically to promote wound healing more effectively. Jiang et al. developed a flexible bioelectronic system for smart bandages that consists of wirelessly powered, closed-loop sensing and stimulation circuits with skin-interfacing hydrogel electrodes that can adhere and detach on demand [69]. The smart bandage system demonstrated the ability to continuously monitor skin impedance (the response of the skin to an electric current, which indicates how healed the wound is) and temperature (increased temperature indicates an infection) and deliver electrical stimulation in response to the wound environment, displaying approximately 25% faster healing and 50% enhancement in dermal remodeling compared to the unstimulated group [69]. Additionally, a bionic artificial skin that incorporates a wireless tactile sensory system allowed for real-time monitoring of pressure and temperature changes, enabling effective wound healing and restoration of tactile function [214]. The wireless sensory system enables effective wound healing and restoration of the tactile function by providing a quantitative analysis of leg movement angle and electromyogram (EMG) signals in response to applied pressures.

#### 5.2.7. Remote Digital Postoperative Wound Monitoring and Machine Learning Applications

Innovative technologies such as a water-modulated biomimetic Hygel E-skin [215] and paper-like battery-free in situ AI-enabled multiplexed (PETAL) sensor patches [166] offer unique solutions for wound monitoring and assessment. These technologies leverage deep learning algorithms, colorimetric sensors, and advanced materials to capture sensory information, provide holistic wound assessment, and enable real-time monitoring of wound healing progress. Additionally, bioinspired cutaneous receptor–perceptual systems [216] and stimulus-responsive wound dressings [217] have been designed to visualize skin physiological status remotely and adjust antibiotic release based on wound conditions, enhancing treatment outcomes.

The implementation of remote digital postoperative wound monitoring using smartphone-delivered tools has revolutionized postoperative care pathways by enabling patients to assess their wounds remotely and report any concerns to clinicians [67]. Machine learning algorithms have also been integrated into artificial skin signal processing to improve signal analysis accuracy and enhance diagnostic capabilities by learning from collected data patterns [198].

Overall, these advancements in wound management technologies showcase the potential for innovative solutions to improve patient outcomes, enhance wound care practices, and revolutionize the field of dermatology and wound healing.

### 5.3. Challenges and Future Perspective on E-Skin for Wound Monitoring and Management

The application of wound dressing in E-skin presents both challenges and exciting prospects for the future. Smart wound dressings with advanced technology are revolutionizing wound care by requiring biocompatible materials, flexibility, adhesion to the skin without feeling foreign, and painless removal. Antimicrobial materials are preferred to reduce infection, which is the biggest problem in wound regeneration. The development of transparent bandages that allow the wound healing process to be seen is also favored. The smart bandage should be resistant to exudate, stable to maintain its functionality, and biodegradable to be environmentally friendly. It should be easy to manufacture and inexpensive to produce. Self-powering and energy-harvesting technologies, as well as wireless communication integration, will enable seamless data transfer for continuous wound monitoring and remote medical applications. A closed-loop feedback system will enable on-demand treatment based on wound monitoring. These needs require the integration of multidisciplinary and integrated technologies into a single smart band. To do this, researchers and innovators can unlock the full potential of E-skin technology in revolutionizing healthcare and improving patient outcomes. Through collaborative efforts and technological advancements, the vision of E- skin as a versatile and effective tool for wound management is within reach.

**Table 4:** Summary of E-skin for wound monitoring and treatment.

Polymer	Composites/ Components	Properties/ Function	Monitoring	Treatment	Ref.
Polyaniline, PVA	PDA decorated AgNPs	Conductive antimicrobial self-healing repeatable adhesiveness	Epidermal sensor	Diabetic Foot Wound Dressings	[182]
P(AM-co-SBMA)	MXene PDA AgNPs	Conductive, antibacterial, biocompatible, self-adhesive, stretchable	Epidermal sensor	Diabetic Wound Healing	[183]
Carboxyl Methyl chitosan (CMCS), phenylboronic acid grafted sodium alginate	MXene nanosheets network catechol-rich Tea polyphenol	Self-healing and shear-thinning properties injectable self-healable, adhesive conductive antibacterial and hemostatic properties	Epidermal sensor electrophysiological signals like electromyogram (EMG) and electrocardiogram (ECG) signals	Hemostatic potential in mouse liver hemorrhage and mouse tail amputation models	[189]
Polyurethane P(MMA-BA)	LM PDA Recombinant spidroin protein	Stretchability pressure sensitivity	Motion Tactile sensing Biochemical sensing	Promoting wound healing	[138]
Gelatin, dextran	Hydroxyapatite curcumin	Antimicrobial antioxidant, pH-responsive color change	pH	Rapid wound healing	[197]
GelMA	CNT, graphene	Breathability, conductivity, stretchability, moisture absorption capacity	Moisture sensor strain sensor	Wound management and monitoring	[201]
PLA/PVP	MXene F sensor (PANI and Ag/AgCl electrode)	Stretchability, bendability, breathability, anti-infection	Wound pH	Photothermal therapy Faster wound healing	[205]
Paper and inkjet printing Binder: ethylcellulose polymer	Oxygen sensing dye Oxygen generation catalyst (KMnO_4_) surfactant	Low-cost Continuous oxygen delivery and sensing Flexible Scalable, biocompatible	Oxygen	Oxygen delivering Increase in oxygen levels to clinically relevant levels.	[206]
Alginate/CaCl_2_	Gentamicin Growth factors Dowex bead conjugated with dye Growth factors	User-friendly colorimetric sensor	pH glucose	Faster wound healing, diabetic wounds	[167]
PDMS	LM heater NFC-integrated circuit PNIPAM-co-AM hydrogel bead antibiotics	Microfluidic channel flexible and wearable on-demand drug release	Temperature humidity	Faster healing of an infected wound	[213]
PEDOT:PSS dry pellet NIPAM/AAM/ MBAA	NFC transponder Ag/AgCl electrode Platinum electrode H_2_O_2_, ascorbic acid	Conductive Flexible Biocompatible Attachable/detachable on demand Delivering electrical stimulation	Temperature Skin impedance	Wound care and healing	[69]
Collagen Fibrin, thrombin	Neural interface electrodes		Pressure temperature	Wound healing and restoring skin tactile function	[214]

Abbreviations: AgNPs (silver nanoparticles); CNT (carbon nanotube); GelMA (Gelatin methacryloyl); LM (liquid metal); NFC (Near Field Communication); NIPAM/AAM/MBAA (N-isopropylacrylamide/acrylamide/N,N’-methylenebisacrylamide); PANI (Polyaniline); Polycaprolactone (PCL); PDA (polydopamine); PDMS (Polydimethylsiloxane); PEDOT:PSS (poly(3,4-ethylenedioxythiophene) polystyrene sulfonate); P(MMA-BA) (poly methyl methacrylate and n-butyl acrylate); PNIPAM-co-AM (Poly(N-isopropylacrylamide-co-acrylamide); Polyvinyl alcohol (PVA).

## 6. Applications and Future Directions

### 6.1. Description and Limitations of Wound Care in Biomimicry

Efforts to improve wound management have continued, with the use of biomimicry technology. This technology has developed over a long period of time through natural selection and evolution. There are several wound management technologies that incorporate biomimicry, including simple designs, biotechnology, medicine, and nanotechnology. Examples of biomimetic technologies include imitating human skin or the biological environment to aid in regeneration, drawing inspiration from the design of animals and plants, and utilizing NPs for various applications. However, despite the promising results and innovative ideas, only a limited number of examples have successfully passed clinical trials or been implemented in real-world scenarios. The development of biomimetic technologies is a complex process that involves various factors. While wound management techniques have shown improved effects, only a few cases have demonstrated multiple functions simultaneously. The use of biomimicry can lead to complicated design and technology, making production difficult. Furthermore, combining technologies that apply the design of various living things into one design has encountered limitations [218]. However, ongoing studies aim to address these limitations, and biomimetic wound management technology remains a promising field. While animal imitation has been extensively explored, the potential of plant-inspired designs and principles has been largely overlooked. By leveraging the strengths of plants, more advanced technology can be developed [219]. Furthermore, the combination and development of various disciplines in wound management technology offer new possibilities, as it allows for the integration of not only existing technologies but also more diverse ones.

### 6.2. Consider the Challenges and Opportunities for Clinical Adoption

Several wound management technologies utilizing biomimetics have been proposed, and the effectiveness of some has been confirmed through clinical trials. A biomimetic nanofibrous membrane based on the properties of the ECM was developed to treat chronic wounds [220]. The nanofibers, composed of poly-L-lactide/gelatin, maintained hydrophilicity with a processing thickness of 0.65 μm and a contact angle of 80.39°. Additionally, it enhanced cell proliferation and reduced bacterial adhesion. When the significant effects confirmed in vitro were applied clinically, the healing rate of chronic wounds was 93.3%. Only 6.7% of these cases required reoperation. Furthermore, 66.7% of patients used only one nanofibrous membrane during wound healing. Bio-inspired skin substitutes, such as Integra^®^ or Matri Derm^®^, are available on the market [221]. Biomimetic wound management technology has shown promising results in clinical trials and is currently being used in the market. This indicates that biomimetic technology has practical applications in wound care.

Mobile phones have proven to be a valuable tool for transmitting demographic, clinical, survey, and progress data to healthcare providers and providing an instant communication channel for timely and reliable advice to those seeking healthcare [222,223]. The World Health Organization (WHO) has defined mobile health (mHealth) as “medical and public health care supported by mobile devices, such as mobile phones, personal digital assistants, wireless devices, and patient monitoring devices.” [224].

A smartphone application has been developed for the early detection of a malignant melanoma [225,226]. The system utilizes a client-server architecture to allow smartphone users to assess the malignancy of skin lesions and distinguish between micro-melanoma and developed skin moles. This provides a promising direction for the early detection of melanoma. The E-skin application on smartphones incorporates scientifically proven algorithms to detect and analyze dermoscopy images for visible signs of skin cancer [225]. SkinScan, SkinVision, and SpotMole are currently available smartphone apps. SkinVision utilizes a machine learning algorithm, while the other two follow the ABCDE rule, which assesses asymmetry, border irregularity, non-uniform color, diameter greater than 6 mm, and changes in size, shape, or color [227]. These devices, along with image storage and analysis technologies, represent a significant advancement in skin cancer diagnosis. However, there is a current issue with the accuracy of the available technologies and the resulting legal liability [227].

During the COVID-19 pandemic, mHealth platforms have been utilized to address the challenges posed by the pandemic [223]. A team at Northwestern University developed a wireless, Bluetooth-connected E-skin device that provides real-time monitoring of talking, breathing, heart rate, and other vital signs [228]. This device has been used to spot symptoms of COVID-19 in front-line health workers and patients.

Studies of wearable devices used to continuously monitor patients and monitor biomarkers other than physical activity (e.g., heart rate, respiratory rate, blood pressure, etc.) have shown promise for monitoring heart rate and sleep in hospitalized patients, but they were often unvalidated in the inpatient setting, and even when validated, measurements were sometimes inconsistent with gold standards [229].

Precision monitoring is closely related to the diagnosis and treatment of patients’ diseases, making the signal accuracy and data reliability of E-skin application a critical issue [230]. Here are other issues that need to be addressed before E-skins can be widely utilized in clinical settings.

First, a practical problem associated with E-skin sensors is current leakage or electromagnetic interference due to insufficient insulation [156]. Second, an E-skin requires a reliable and efficient power supply. While research has focused on self-powered E-skins based on TENGs that convert mechanical energy into electrical power, optimizing these systems for consistent performance is a challenge [231]. Third, E-skins need to be able to withstand varying degrees of stretching and bending without loss of functionality when attached and used on a daily basis. This requires them to be durable and flexible to prevent breakage during use [232]. Fourth, since E-skins are intended to be attached to the body, they must be biocompatible to prevent adverse effects such as allergic reactions or inflammation when in contact with human skin [232]. Fifth, despite advances at the lab level, mass production of prototypes remains a challenge. Efficient manufacturing processes for consistent products, simplification of the manufacturing process, and a reduction in production costs are key factors for commercialization [233]. Sixth, one of the adoptions of AI-powered models in healthcare is the lack of high-quality medical data, which can result in inaccurate outcomes. Additionally, data privacy, availability, and security pose potential limitations to the application of AI in this field. It also requires the consideration of AI-powered models’ mistakes [234]. Seventh, E-skin devices need regulatory clearance for medical applications [235,236]. To overcome the regulatory hurdles, the limitations presented above need to be addressed as well.

### 6.3. Propose Future Directions for Research and Development

Biomimetics is a promising approach to wound care technology and has potential in treating other diseases and symptoms. Among the prospects, several are worthy of note.

(1) Biomimetic wound dressings have shown great promise in promoting wound healing. The traditional approach to wound dressings has limitations in supporting complex wound healing processes, as they primarily focus on sealing the wound, absorbing exudate, and maintaining a moist environment. However, by incorporating ECM components like collagen, glycosaminoglycan, or hyaluronic acid into traditional dressings, it can actively promote epithelialization, angiogenesis, and collagen deposition, thereby enhancing the overall wound healing process [90,237,238].
(2) Biomimetics presents a promising approach to wound care technology and has the potential to extend its benefits to the treatment of other diseases and conditions. The field of biomedicine can benefit greatly from mimicking components or micro- and nanostructures found in various living organisms such as animals, plants, and humans [239]. Materials that utilize these biomimetic components or structures offer numerous advantages, including biocompatibility, biodegradability, targeting efficiency, low toxicity, and antioxidant and anti-inflammatory properties. These biomimetic materials have proven effective in wound healing technologies and show promise in the treatment of a variety of diseases, particularly cancer [119,240].
(3) Traditional wound dressings lack the ability to provide real-time information about wound conditions, resulting in missed opportunities to adjust treatment. Therefore, there is a need for versatile smart E-skin patches that can accurately monitor wound conditions and accelerate wound healing. For this, E-skin materials are required to possess properties such as elasticity, self-healing capabilities, biocompatibility, skin-like softness, and the ability to generate electrical signals for rapid sensory transmission [241,242]. Researchers are exploring new materials and structural designs to achieve these properties using hydrogels, liquid metals, conductive polymers, and nanomaterials [243]. Scientists at Stanford University have made a significant breakthrough in synthetic skin technology. They developed a multi-layered thin film sensor that heals by auto-realignment, closely resembling the natural healing process of our skin [244].
(4) The advancement of E-skin, with its soft, stretchable, biocompatible, and adhesive properties, enables continuous health monitoring as a wearable device that adheres well to the body due to its soft and flexible nature [245]. In addition, for continuous signal sensing and monitoring, E-skin devices need to operate wirelessly and continuously [156,246]. Energy self-generation, storage, and power efficiency are crucial to provide enough power without compromising comfort or fit. Furthermore, advances in E-skin’s strain sensors can act as a sensitive touch interface or even detect gestures and movements, enabling a seamless human–machine interface. This human–machine interface can be applied to sensitive health monitoring [247,248]. Wearable self-powered sensors can be used to monitor vital signs (heart rate, temperature, blood pressure, etc.), detect skin conditions such as pressure ulcers and diabetic patients, track wound healing progress, and so on, for the healthcare of the elderly population and patients with cardiovascular disease and diabetes [248].
(5) Future research will focus on the development of E-skins that closely mimic human skin in terms of functionality and properties, including mechanoreceptors, softness, flexibility, self-healing ability, and environmental adaptability, and even develop E-skins that can outperform human skin in certain areas [249,250]. Meanwhile, making artificial skin that acts like real skin is hard due to material issues, but using AI and machine learning could help find and improve materials, making the skin better and safer [251]. Future developments in E-Skin sensors will include increased accuracy, improved battery life, and integration of more sensors for comprehensive health monitoring. Integrating AI using machine learning algorithms into the E-skin for independent data analysis can help us to understand the user’s personalized health profile, enabling the creation of precise personalized treatment plans. This will enable the creation of personalized and accurate monitoring and treatment plans. Additionally, drug delivery mechanisms can be integrated directly into E-skins for targeted therapy. It is important to consider the growing concern for the environment, leading to the development of sustainable and recyclable E-skins as an important future direction.

## Data Availability

Not applicable.
